# Autophagic Control of Skin Aging

**DOI:** 10.3389/fcell.2019.00143

**Published:** 2019-07-30

**Authors:** Leopold Eckhart, Erwin Tschachler, Florian Gruber

**Affiliations:** ^1^Research Division of Biology and Pathobiology of the Skin, Department of Dermatology, Medical University of Vienna, Vienna, Austria; ^2^Christian Doppler Laboratory for Biotechnology of Skin Aging, Vienna, Austria

**Keywords:** skin, autophagy, aging, epidermis, keratinocytes, hair, sweat gland, melanocytes

## Abstract

The skin forms the barrier to the environment. Maintenance of this barrier during aging requires orchestrated responses to variable types of stress, the continuous renewal of the epithelial compartment, and the homeostasis of long-lived cell types. Recent experimental evidence suggests that autophagy is critically involved in skin homeostasis and skin aging is associated with and partially caused by defects of autophagy. In the outer skin epithelium, autophagy is constitutively active during cornification of keratinocytes and increases the resistance to environmental stress. Experimental suppression of autophagy in the absence of stress is tolerated by the rapidly renewing epidermal epithelium, whereas long-lived skin cells such as melanocytes, Merkel cells and secretory cells of sweat glands depend on autophagy for cellular homeostasis and normal execution of their functions during aging. Yet other important roles of autophagy have been identified in the dermis where senescence of mesenchymal cells and alterations of the extracellular matrix (ECM) are hallmarks of aging. Here, we review the evidence for cell type-specific roles of autophagy in the skin and their differential contributions to aging.

## Introduction

Changes of the skin belong to the most recognizable signs of aging. Accordingly, skin aging is a major area of interest for cosmetic and skin care industries. From the medical viewpoint, aging of the skin is associated with health problems including increased skin fragility, delayed wound healing and the increased occurrence of skin cancers, the most abundant types of malignancies in humans. The prevention and management of skin aging depends on a thorough understanding of the aging process in general, which can be defined as the time-dependent decline in tissue and organismal functions (Leidal et al., 2018), and on the understanding of the function and interplay of the unique cell types that build the skin. For a long time it has been recognized that the rate of skin aging is determined by intrinsic and extrinsic drivers, but only recent advances in skin gerontology have helped to dissect the molecular and cellular processes that underlie the aging of the skin (Gilchrest and Krutmann, 2006; Chang, 2016; Ghosh and Capell, 2016; Botchkarev, 2017; Rinnerthaler and Richter, 2018; Wang and Dreesen, 2018). Several of the aging processes are triggered or enhanced by the presence of damaged molecules and organelles within cells, and their turnover is controlled partly by autophagy. Besides proteostasis and organelle maintenance, other factors that are accepted hallmarks of aging (López-Otín et al., 2013), such as nutrient sensing and genomic instability are under the control of or elicit the activation of autophagy, making autophagy a major counter-regulatory process that supports skin homeostasis and healthy aging.

Autophagy is a process of cellular self-digestion by delivering cytoplasmic material to the lysosome for breakdown. Three pathways of autophagy can be distinguished mechanistically, i.e., macroautophagy, microautophagy, and chaperone-mediated autophagy. Macroautophagy is controlled by autophagy-related proteins (ATGs) and depends on the sequestration of material within double-membraned vesicles (autophagosomes) in the cytoplasm. These vesicles fuse with lysosomes to form autophagolysosomes in which the cargo is degraded by lysosomal enzymes. Breakdown products are released from lysosomes and subsequently are utilized for catabolic processes or energy production. Microautophagy is characterized by the invagination of the lysosomal membrane in yeast, whereas in mammals it involves the invagination of the late endosomal membrane to trap cytoplasmic material which is then degraded either in late endosomes or in lysosomes after endosomal-lysosomal fusion (Tekirdag and Cuervo, 2018). Chaperone-mediated autophagy relies on the cytosolic chaperone hsc70 for substrate targeting to the lysosome. Proteins that contain a consensus pentapeptide motif are bound by hsc70, unfolded and translocated into the lysosomal lumen in a lysosome-associated membrane protein type 2A (LAMP2A)-dependent manner (Tekirdag and Cuervo, 2018). For further details of the mechanisms of autophagy, the reader is referred to many excellent reviews (Rubinsztein et al., 2011; Boya et al., 2013; Galluzzi et al., 2017; Hansen et al., 2018; Levine and Kroemer, 2019). In the present review of autophagy in the skin, the term “autophagy” is equivalent to “macroautophagy” unless stated otherwise.

Here, we review the features of skin aging at the levels of the tissue and the cells and then describe the contributions of autophagy to the control of skin aging. We propose a concept in which aging is driven by changes in three categories of cells with different dependencies on autophagy.

## Skin Aging at the Tissue Level

**FIGURE 1:**
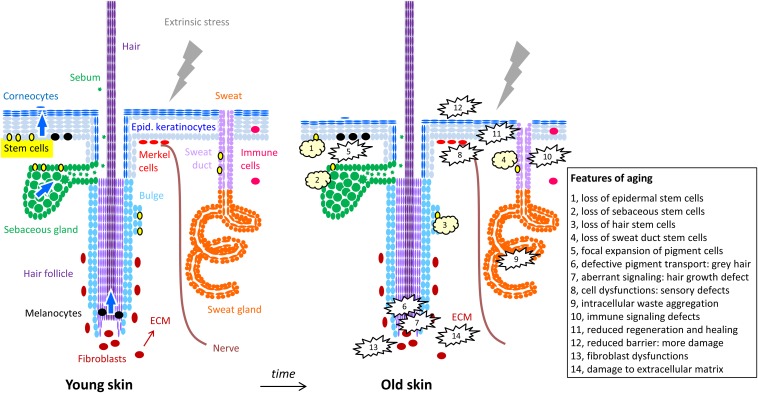
Cells of the skin and their changes during aging. The structure of the skin is schematically depicted with oval symbols representing cells. Molecular and cellular features of aging are indicated in the right panel and in the box. For simplification, the diversity of immune cells and dermal cell populations are not depicted here.

The skin is a composite organ consisting of a layer of subcutaneous fat tissue, the mesenchymal dermis and the outermost epithelial layer, the epidermis (McGrath, 2005). In addition, there are skin appendages, i.e., hair, sebaceous glands, sweat glands and nails, which are derived through the differentiation of epithelial cells in cooperation with mesenchymal cells. These skin appendages are anchored mainly within the dermis (McGrath, 2005; Figure 1, left panel).

The subcutaneous fat tissue functions both as insulation against temperature fluctuations and as energy storage, and is composed mostly of lobules of adipocytes which are surrounded by a lattice of collagen septae and blood vessels. It provides the link of the skin with the underlying muscles and fasciae. The dermis consists mostly of extracellular matrix (ECM) with collagen, elastin, and glycosaminoglycans as the principal components, all of which are produced by fibroblasts, the most abundant cell type within the dermis. The dermal ECM provides the skin with both strong mechanical resistance and elasticity (Bruckner-Tuderman, 2012). Embedded into the dermis are networks of blood and lymphatic vessels, which supply oxygen and nutrients to the skin, regulate the body temperature, and serve as highways for immune cell trafficking. The vast web of dermal nerve endings reaches as far as into the epidermis and transmits sensorial perceptions such as temperature, touch, pain and itch to the central nervous system (McGlone and Reilly, 2010). Adding to the complexity of this tissue, it harbors a multitude of resident immune cells including mast cells, tissue macrophages and professional antigen presenting cells (McGrath, 2005).

Attached to the dermis by specialized proteins of the basement membrane, the epidermis forms the outermost skin layer. It is a stratified epithelium consisting mainly (95% of all epidermal cells) of keratinocytes, which undergo terminal differentiation and form the stratum corneum, which serves as the ultimate barrier against the environment (Eckhart and Zeeuwen, 2018). Besides keratinocytes, the epidermis contains regularly dispersed Langerhans cells, the remotest antigen presenting cells of the immune system, and melanocytes, which produce the UV protective melanin and transfer it to the adjacent keratinocytes thereby determining also the color of the skin and hair (McGrath, 2005).

In contrast to internal organs, the signs of skin aging are immediately recognizable also to non-medically trained observers. Most obvious are the occurrence of facial wrinkles which are either the consequence of repeated activity of the underlying muscles during facial expression, or tissue slackening as a consequence of loss of elastin and collagen fibers. Tissue slackening manifests with a loss of the facial oval and sagging of the upper eyelids. In addition, pigment irregularities of sun-exposed skin are a hallmark of aging skin (Guinot et al., 2002).

When analyzing skin aging, one has to take into account that apart from intrinsic aging which proceeds in all organs and is genetically determined, skin aging is also strongly modified by extrinsic factors such as chronic sun exposure and life style habits in particular cigarette smoking (Guinot et al., 2002). Intrinsic aging is characterized primarily by a progressive loss of skin tissue, i.e., a thinning of all skin layers. The subcutaneous fat pad decreases with age and so do the components of the dermal ECM. Functionally, the loss of the fat pad results in an increased sensitivity to both hot and cold temperature whereas the loss of the dermal ECM makes the skin more fragile and prone to wounding. Within the epidermis the turnover of keratinocytes is reduced resulting in a thinning of the epidermis and a reduced capacity to rapidly restore the skin barrier after barrier breaks. As to the skin appendages, hair becomes thinner and on the scalp terminal hair follicles are gradually miniaturized (Fernandez-Flores et al., 2019). Most obvious, the natural hair color is lost due to a reduced transfer of pigment from follicular melanocytes to hair keratinocytes and air inclusions into the hair shaft (Fernandez-Flores et al., 2019). Sebum production decreases with age in particular in women after menopause. Similarly, sweat secretion decreases with aging (Dufour and Candas, 2007). Furthermore, chronic pruritus or itch is common problem in the elderly, indicating an age-related conversion of touch to itch sensation due to loss or defects of Merkel cells (Feng et al., 2018).

In contrast to intrinsic or chronological aging, photoaging concerns only body regions which are chronically exposed to sunlight or to artificial sources of ultraviolet (UV) radiation (Kligman and Balin, 1989; Yaar and Gilchrest, 1998; Gilchrest, 2013). The difference between intrinsic aging and photoaging can most easily be observed in a given individual when comparing UV-exposed skin sites such as the outer forearm to non-exposed sites such as the inner upper arm. Whereas intrinsic aging is characterized by a loss of ECM, photoaged skin contains abundant elastin and collagen fibers, however, they are not arranged like in non-exposed skin but are fragmented and disorganized due to the action of UV-induced proteases (Gilchrest, 2013). In fact the appearance of photoaged skin has been likened to scar tissue (Fisher et al., 1997). In addition, photoaged skin displays very distinct pigment irregularities referred to as age spots and, on the histological level, immune cell infiltrates including mast cells, resulting in a subclinical chronic inflammation. At the molecular level, UV-signature mutations can be detected mainly in keratinocytes and melanocytes long before they become clinically relevant by causing actinic keratoses, basal cell carcinomas or melanomas (Jonason et al., 1996).

## Skin Aging at the Cellular Level: Stem Cells, Short-Lived Epithelial Cells and Long-Lived Differentiated Cells

**FIGURE 2:**
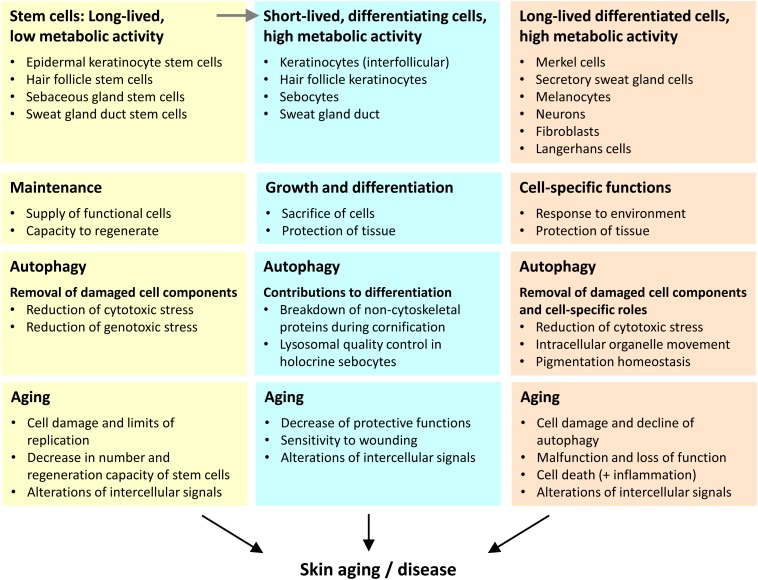
Three categories of cells play different autophagy-dependent roles in skin aging. We propose that the aging of the skin is driven by changes in the distinct types of skin cells that we categorize according to lifetime (inversely correlated to turnover) and differentiation (execution of specific function within the tissue context). The main roles of autophagy and aging-associated changes are summarized for each cell category.

Aging is a complex phenomenon which includes many effects at the systemic level but also critical changes at the level of isolated system components such as DNA, cells, and tissues. Here, we put forward the hypothesis that drivers of aging mediate their effects differentially on the various types of cells in the skin and perhaps also in other tissues. We propose to distinguish three categories of cells that change during aging in different ways (Figure 2).

First, stem cells have a long lifetime and relatively low metabolic activity. Within the epidermis the stem cells themselves do not directly contribute to the epidermal barrier, but they give rise to differentiating daughter cells that achieve this function. A decline in the number and activity of stem cells is both driver and marker of skin aging. Of note, epithelial structures with a high cell turnover, such as epidermal keratinocytes strongly depend on stem cells during homeostasis whereas other skin compartments require stem cells predominantly or only during wound repair.

Second, there are cell types that differentiate and undergo fast turnover when they fulfill their function. The metabolic activity of these cells is very high. Examples for this category are epithelial cells of the interfollicular epidermis, sebaceous glands, hair follicles, and the duct of the sweat gland. The maintenance of function of these differentiating cells requires their continuous derivation from stem cells.

Third, differentiated skin cells of diverse developmental histories fulfill specific functions over prolonged periods of time or even during the entire life of an organism. Typically, these cells have significant metabolic activity over long times. Examples of this cell category are neurons, melanocytes, Merkel cells, secretory cells of sweat glands, and fibroblasts.

## Skin Aging at the Cellular Level: Differential Roles of Autophagy in Three Categories of Cells

The skin provides several examples to illustrate the two main interactions between autophagy and aging: (1) Autophagy decreases the rate of aging and (2) the activity of autophagy declines during aging. Autophagy suppresses aging in a cell-autonomous manner by maintaining intracellular homeostasis and in a non-autonomous manner by contributing to various cell features that protect other cells. For instance, autophagy supports the differentiation of epithelial cells which allows them to protect other cells against external noxae (Velarde, 2017). Since autophagy achieves the removal and recycling of intracellular material only to a certain extent, potential toxic cell components and dysfunctional lysosomes tend to accumulate during the life-time of cells. Some of the compromised cells succumb to cell death whereas others remain alive but lose their capacity to execute intracellular processes, including autophagy, with full efficiency. Loss and dysfunction of cells manifest in aging.

Autophagy was reported to play various roles in the homeostasis and stress response of skin cells that were reviewed previously (Sukseree et al., 2013a; Sil et al., 2018). Most recently, the roles of autophagy in immune cells of the skin were characterized (Das et al., 2019). For the main types of skin cells, the roles of autophagy and their relevance for aging will be discussed below. According to the above categorization of skin cells, autophagy has different sensitivities to aging-associated processes and different roles in driving processes that enhance skin aging (Figure 2).

**FIGURE 3:**
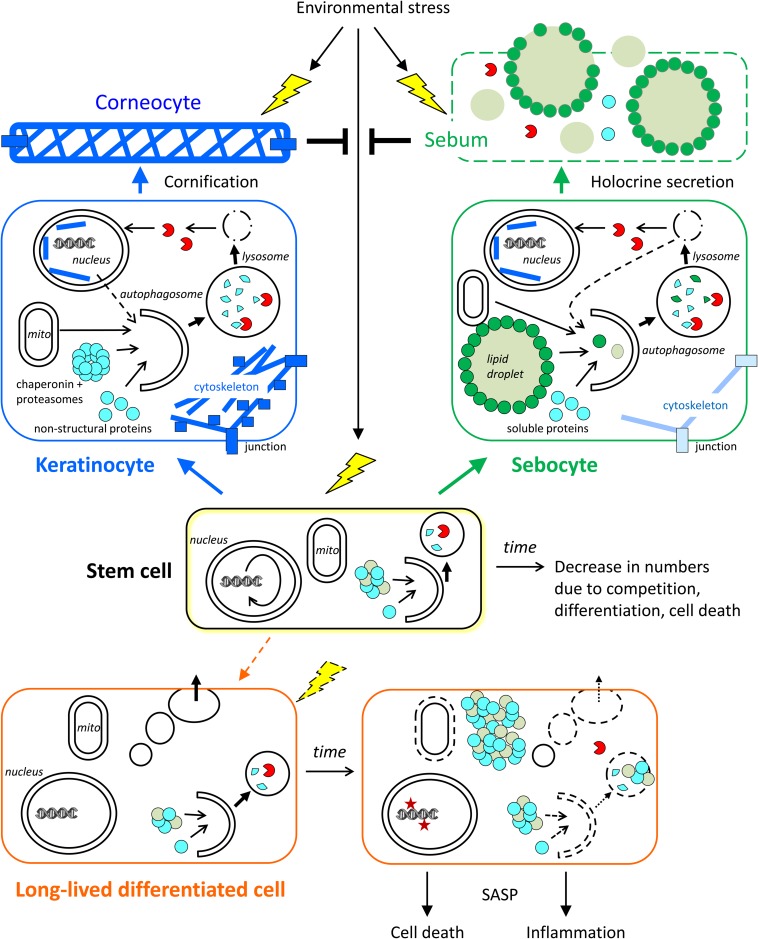
Roles of autophagy in skin cells. The contributions of autophagy to cornification, holocrine secretion, and homeostasis of stem cells and long-lived differentiated cells (exemplified by a sweat gland secretory cell) are schematically shown. Time-dependent changes, such as a decline of autophagy, affect mainly stem cells and long-lived differentiated cells. Short-lived keratinocytes and sebocytes are continuously generated from stem cells and then differentiate rapidly. Autophagy contributes to the formation of corneocytes and sebum, which protect the living cells of the skin against stress factors from the environment. The conversion of stem cells into (short-lived) differentiating keratinocytes and sebocytes occurs throughout life while the conversion of stem cells into long-lived differentiated secretory cells occurs rarely in adult life. Note that specialized stem cells exist in the interfollicular epidermis and in skin appendages whereas only one stem cell is shown in this simplified schematic drawing.

(1) Long-lived and mostly quiescent stem cells require autophagy for intracellular homeostasis and for continuous ability to supply functional progeny cells (García-Prat et al., 2016; Boya et al., 2018). Inherent decline or exogenous suppression of autophagy leads to stem cell loss by competition, differentiation, or cell death (Figure 3).
(2) In short-lived differentiating cells, autophagy also contributes to intracellular homeostasis, however, autophagic activity needs to be maintained only over a short time for these cells to be functional. External and internal factors have little time to impair autophagy during differentiation and consequently an aging-associated decline of autophagy is less likely to occur in these cells. Nevertheless, autophagy defects can be inherited from the long-lived precursor cells (stem cells) and potentially compromise processes such as the defense against microbes, the release of cytokines, and most importantly, the protection against stress factors from the environment (Figure 3).
(3) In long-lived differentiated cells, autophagy contributes to the maintenance of cell survival and function. A decrease of autophagy leads to the accumulation of damaged or even toxic components and/or energy crisis. These disturbances of intracellular homeostasis impair the processes essential for cell functions and eventually lead to a loss of these cells (Figure 3).

Importantly, the process of aging is not fully understood at present and several new concepts of aging drivers have not yet been investigated for their potential interaction with autophagy. For instance, stem cell competition for adherence to the basement membrane depending on proteolytic decrease of COL17A (Settembre et al., 2018; Liu et al., 2019) has emerged as an important process that contributes to aging of skin epithelia. Another emerging mechanism with an impact on aging is the immune surveillance of senescent cells (Ovadya et al., 2018). The potential roles of autophagy in proteolysis and antigen processing during the above-mentioned process will be interesting topics of future investigations.

## Autophagy in the Different Cell Types of the Skin

The literature on roles of autophagy in the skin is largely organized into reports that focus on cell types of specific developmental origins. Here, we review these reports in separate sections, divided in epithelial and non-epithelial cell types. Non-epithelial skin cells are largely long-lived and differentiated, i.e., category 3 cells as defined in the previous sections of this article, with regard to autophagy and skin aging. Among the non-epithelial skin cells, there are also stem cells (category 1), but the changes in long-lived differentiated cells are considered the main contributors to aging. Epithelial cells of the skin include only a quantitatively minor portion of long-lived differentiated cells (category 3), such as Merkel cells and secretory cells of the sweat glands, whereas the main portion of epithelial cells are short-lived, differentiating cells (category 2), such as keratinocytes of the interfollicular epidermis, hair follicle keratinocytes, sebocytes, and sweat duct cells. The replenishment of the differentiating epithelial cells strongly depends on epithelial stem cells (category 1) (Figure 2). The contributions of autophagy to the maintenance and functions of stem cells and differentiated cells are discussed in detail below.

## Autophagy in Epithelial Cells of the Skin

### Autophagy in Epidermal Keratinocytes

Keratinocytes are the main cell type of the epidermis where they represent more than 95% of the total cell population and autonomously establish a stratified epithelium. Keratinocyte stem cells reside in the basal layer of the epidermis and in special locations of epidermal appendages, such as the bulge of hair follicles and the duct of sweat glands (Figure 1). Proliferating keratinocytes, so-called transit amplifying cells, require attachment to the basement membrane and therefore are also restricted to the basal layer. Upon detachment from the basement membrane, keratinocytes activate a genetically controlled terminal differentiation program that leads to the enhancement of the cytoskeleton as the cells move toward the epidermal surface and ultimately to cornification (Eckhart et al., 2013; Figures 1, 3).

Due to the fast turnover of keratinocytes, keratinocyte aging manifests mainly in changes of stem cell numbers and functions. In addition, changes in epidermal structure and stress responsiveness decrease the function of the epidermis as protection against pro-aging stress factors (Velarde, 2017). There are multiple extrinsic drivers of aging including UV irradatiation, exposure to environmental toxins, and disruptions of the barrier by low humidity, detergents, and microbes. Intrinsic drivers of aging are stem cell competition, oxidative stress due to mitochondrial dysfunctions, and others (Gilchrest and Krutmann, 2006). Keratinocyte autophagy is suppressed in carriers of a mutation in *AP1S3*, leading to accumulation of p62/sequestosome-1 (Sqstm1) and inflammation (Mahil et al., 2016).

Autophagy is active both in proliferating and differentiating keratinocytes of human and mouse epidermis (Haruna et al., 2008; Rossiter et al., 2013; Sukseree et al., 2013b; Yoshihara et al., 2015). The programmed degradation of the nucleus and mitochondria during cornification is reminiscent of autophagy and, indeed, there is correlative evidence for a role of the autophagy machinery in organelle degradation within the granular layer of the epidermis (Akinduro et al., 2016). Keratinocyte-specific deletion of *Atg5* and *Atg7* in mice led to thickening of the stratum corneum but did not block the removal of the nucleus in cornifying keratinocytes (Rossiter et al., 2013; Sukseree et al., 2013b).

### Roles of Autophagy in Keratinocyte Stem Cells

Autophagy supports the maintenance of stem cells (Cho et al., 2019). Both self-renewal and quiescence of stem cells were reported to require autophagy (García-Prat et al., 2016; Wang et al., 2018). In keratinocyte stem cells, the expression of autophagy-regulatory genes changes during the circadian rhythm and during aging (Solanas et al., 2017). Suppression of autophagy by epithelium-specific deletion of *Atg5* or *Atg7* can maintain a functional skin barrier during aging, suggesting that autophagy is not critically required for epidermal stem cell maintenance under non-stressed housing conditions of mice (Rossiter et al., 2013; Sukseree et al., 2013b). Interestingly, mice with epithelium-specific *Atg7* knockout developed a thicker epidermis and larger sebaceous glands than wild-type mice and thereby resembled other mouse lines with keratinocyte hyperproliferation (Rossiter et al., 2013, Rossiter et al., 2018). Quantitative studies of stem cell numbers and functions at different age of mice are needed to explore the role of autophagy in epidermal stem cells.

### Roles of Autophagy in Stress Resistance of Epidermal Keratinocytes

Autophagy has been implicated in the response of keratinocytes to various types of stress, most of which accelerate skin aging. The best characterized environmental stress on skin is UV radiation which induces oxidation of skin molecules and damages the genetic material. UVB irradiation of the skin induces DNA damage and inflammation (Gilchrest, 2013). Autophagy improves DNA damage recognition by nucleotide excision repair (Qiang et al., 2016) and reduces inflammation and consequently also inflammation-enhanced tumorigenesis of the epithelium (Qiang et al., 2017). However, it has also been reported that autophagy facilitates tumor development under genotoxic stress via suppressing p62-mediated p38 activation and promotion of cell survival (Qiang et al., 2013).

UVA causes oxidation of phospholipids in keratinocytes, but it also induces autophagy which promotes the removal of these oxidation products in epidermal keratinocytes (Zhao et al., 2013). Suppression of Atg7-dependent autophagy increased DNA damage in response to UVA (Song et al., 2017). UVA irradiation also activates transcription factor EB (TFEB) and upregulates expression of p62 as well as cyclooxygenase-2 (COX-2), a prostaglandin synthase implicated in skin cancer development (Sample et al., 2017). *In vitro* studies suggest that the levels of oxidative damage and autophagic activity determine whether senescent keratinocytes undergo programmed cell death or evade senescence with potential neoplastic activity (Deruy et al., 2014). These findings emphasize that cellular senescence, i.e., a state of arrested proliferation and altered metabolism (Muñoz-Espín and Serrano, 2014), is not only a driver of aging but also a cancer-preventive mechanism and therefore should not be regarded as entirely detrimental. In summary, the roles of autophagy in the stress responses of keratinocytes are complex and pharmaceutical modulation of autophagic activity in the epidermis should be evaluated in both homeostatic and stress conditions (Tschachler and Eckhart, 2017).

### Autophagy in Nail Keratinocytes

Keratinocytes of the nail unit differentiate and cornify to become components of the nail. Genetic suppression of autophagy does not abolish growth of nails during aging (Sukseree et al., 2013b; Jaeger et al., 2019), suggesting that autophagy is dispensable for the maintenance of nail epithelial stem cells. However, the conversion of nail keratinocytes into regular cornified nail requires autophagy. Murine nails have been successfully used as a model to decipher the role of autophagy in cornification (Jaeger et al., 2019). Proteomic comparison of nails formed by autophagy-competent and by autophagy-deficient keratinocytes showed numerous differences that suggest an active participation of autophagy in cornification and a broad substrate specificity of cornification-associated autophagy. Deletion of *Atg7* in keratinocytes of the nail unit and other epidermal compartments led to a decrease in the content of keratins and keratin-associated proteins and to a concomitant increase of non-cytoskeletal proteins, including enzymes and proteins with regulatory functions. Most prominently, components of multi-protein molecular machines, such as ribosomes, chaperonins and proteasomes, accumulated upon suppression of autophagy (Jaeger et al., 2019). It appears very likely, but remains to be experimentally confirmed that autophagy plays equivalent roles in the cornification of keratinocytes in hair follicles and interfollicular epidermis (Figure 3).

### Autophagy in Hair Follicle Keratinocytes

Hair is formed by a special type of cornification of keratinocytes in which essentially the entire cell is filled with cross-linked keratin filaments, and hair keratinocytes are stably interconnected to form a long fiber. The growth of hair requires a cylindrical architecture of follicles ensheathing the hair fiber. Growth of hair depends on proliferation and differentiation of keratinocytes which occur at the bottom of the follicle. At this site melanocytes deliver pigment into hair and dermal fibroblasts play important regulatory roles (Figure 1). The growth of hair is terminated when the hair fiber reaches a certain, hair type-dependent length, the fiber is shed and a new cycle of hair growth is initiated. A recent report showed that autophagy is active during hair growth in an organ culture of human scalp hair follicles and inhibition of autophagy promotes hair follicle regression (Parodi et al., 2018).

A decline in hair growth and progressive loss of active terminal hair follicles are characteristic for aging. Recently, it was shown that hair follicle stem cell aging causes the stepwise miniaturization of hair follicles and eventual hair loss in a process that that depended on the proteolysis of type XVII collagen (Matsumura et al., 2016). The cyclic growth and shedding of hair requires a complex communication between epithelial and mesenchymal cells. While autophagy is active in hair keratinocytes (Parodi et al., 2018) and mesenchymal cells surrounding hair follicles (Nicu et al., 2019), it remains to be determined whether and how autophagy contributes to the signaling between different cells of hair follicles.

Recently, small molecule activators of autophagy, such as alpha-ketoglutarate and alpha-ketobutyrate, were reported to induce a switch from telogen (the quiescent phase of the hair cycle) to anagen (the phase of active hair growth) in mice (Chai et al., 2019). Similarly, rapamycin and metformin stimulated growth of hair and their effects could be blocked by autophinib, an inhibitor of autophagosome formation (Chai et al., 2019). Studies in mouse models of alopecia and in humans are needed to determine whether the pharmacological activation of autophagy is able to counteract hair loss.

### Autophagy in Sebaceous Glands

Hair follicles are invariably associated with sebaceous glands that release sebum into the upper hair canal. The sebaceous gland is composed of acini with proliferating cells attached to the basement membrane and differentiating and lipid-accumulating cells being passively moved to the interior where they disintegrate to release a mix of lipid and other cell content into the sebaceous duct (Figure 1). This process of holocrine secretion involves degradation of the nucleus and cytoplasmic changes that are poorly characterized except for the massive accumulation of large lipid droplets (Figure 3). Suppression of autophagy by either deletion of *Atg7* or *Atg5* leads to premature breakdown of the nucleus in differentiating sebocytes (Sukseree et al., 2013b; Fischer et al., 2017). The current hypothesis is that autophagy contributes to the stabilization of lysosomes or to the removal of defective lysosomes in normally differentiating sebocytes, whereas lack of these maintenance and repair mechanisms allows defective lysosomes to trigger cell death that involves degradation of nuclear DNA by the lysosomal endonuclease DNase2 (Fischer et al., 2017).

### Autophagy in Sweat Glands

Sweat has an essential function in thermoregulation of human skin. It is produced by secretory cells and reaches the skin surface via the duct of sweat glands. These glands are developmentally derived from K14-positive epithelial cells but most cells of the mature sweat gland reduce expression of K14 and upregulate other keratins such as K18 in the secretory portion of the glands. The cells of the secretory portion of the sweat gland do not proliferate whereas cells of the duct proliferate to maintain a connection to the surface of the constantly renewing epidermis (Lu and Fuchs, 2014). Aging of human sweat glands is characterized by the accumulation of lipofuscin, an autofluorescent mixture of oxidized proteins and lipids, in secretory sweat gland cells (Cawley et al., 1973; Sukseree et al., 2018a; Figure 1).

Deletion of *Atg7* in skin epithelial cells of the mouse, in which sweat glands are restricted to toe and foot pads, led to accumulation of p62 in secretory cells of sweat glands but not in eccrine duct epithelial cells (Sukseree et al., 2018a). The size and numbers of p62 aggregates increased with age. Moreover, Atg7-deficient glands showed a decrease in cellularity and an aberrant widening of the lumen. The density of functional sweat glands was significantly decreased at around 1 year of age (Sukseree et al., 2018a). Thus, autophagy is essential to suppress the accumulation of p62 during normal aging of long-lived sweat-secreting cells and to maintain the function of sweat glands (Sukseree et al., 2018a). These data should be followed up by studies of autophagic activity and its possible negative correlation with the abundance of lipofuscin deposits in human sweat glands.

### Autophagy in Merkel Cells

Merkel cells are sensors of soft touch in the epidermis and hair follicles. They differentiate from epithelial cells that express keratin K5/K14 and switch to a K8/K18-dependent cytoskeleton. Merkel cells reside in the basal layer where they are either organized in touch domes or in circular arrangement in the upper part of the hair fiber (Sukseree et al., 2018b). Upon physical stimulation, they release serotonergic vesicles at synapses with nerve fibers that transmit the signal further (Figure 1). It was hypothesized that autophagy is active in Merkel cells to degrade a fraction of serotonergic vesicles and thereby to control the strength of signaling at serotonergic synapses (Sukseree et al., 2018b). The sensation of soft touch is important for spatial awareness and communication. Aging and some diseases are associated with the pathologic sensation of innocuous mechanical stimuli as itch, a phenomenon known as alloknesis (Feng et al., 2018).

In *Atg7^*f/f*^ K14-Cre* mice (Sukseree et al., 2012), the deletion of *Atg7* led to marked accumulation of p62 in Merkel cells, suggesting a particular requirement for autophagy in these cells (Sukseree et al., 2018b). Merkel cells have a longer life time than keratinocytes, which allows stronger accumulation of p62. Based on the studies in mouse models (Feng et al., 2018; Sukseree et al., 2018b), the potential link between the role of autophagy in Merkel cell homeostasis and aging-associated defects of touch sensation represents a promising topic of future research.

### Autophagy in Non-epithelial Cells of the Skin

Besides the quantiatively predominant epithelial cells, a developmentally and functionally diverse set of other cells including melanocytes, fibroblasts, Langerhans cells, Schwann cells, and neurons reside in the skin. These cells have in common low turnover and long lifetime which predisposes them to the negative effects of declining autophagy during aging (Figure 2). Melanocytes are the most studied non-epithelial skin cells with regard to roles of autophagy. Autophagy in fibroblasts, nerves, endothelial cells, and immune cells has been studied mainly in other organs than the skin as well as *in vitro*.

### Autophagy in Melanocytes

Melanocytes of the skin produce melanin and deliver it to neighboring keratinocytes that reside either in hair follicles, thus determining hair color, or in the interfollicular epidermis to determine complexion. Aging is associated with the changes of hair color, pallor and appearance of age spots (lentigo senilis), indicating that the function of melanocytes changes with age (Yaar and Gilchrest, 2001). The most important effect of melanocyte dysfunction in aging is graying of the hair. The follicular melanocytes which supply pigment to the hair respond to signals of the hair growth cycle, and their age-related depletion and change in shape (Itou, 2018) together with an increase in redox stress are implicated in age-related dysfunction of the hair follicle pigmentary unit (Tobin, 2015).

The incidence of melanocyte-derived cancers is generally correlated with increasing age, however, malignant melanoma is also relatively frequent in young adults. Nevus cell nevi, commonly known as moles, show age-related variations in shape and appearance (Zalaudek et al., 2006; Schäfer et al., 2006). Non-proliferating melanocytes in nevi show signs of cellular senescence (Tran and Rizos, 2013), nevertheless one third of melanomas derive from nevi. Current models hypothesize that mTOR signaling, which is linked to the control of autophagy, plays a role in the mechanism by which the senescent growth arrest is overcome (Damsky and Bosenberg, 2017). As the following recent studies of melanocytes indicate, autophagy may affect age-related changes in melanogenesis, melanosome transfer, melanocyte cell death, melanocyte redox stress control, melanocyte proliferation rate, melanocyte senescence and inflammatory signaling.

### Roles of Autophagy in Melanogenesis, Melanosome Transfer, and Pigmentation

Proteins of the autophagic machinery facilitate the movement of developing melanosomes within the melanocytes on microtubules and actin filaments (Ramkumar et al., 2017). The melanosomes are transferred to keratinocytes in which autophagy limits the accumulation of transferred melanin (Murase et al., 2013, 2016). Interestingly, mutations in the autophagy gene *EPG5* cause a recessive neurodevelopmental multisystem disorder, Vici syndrome, which is associated with hypopigmentation (Cullup et al., 2013).

Our group has observed mild but age-correlated effects on pigmentation in mice devoid of Atg7-dependent autophagy in melanocytes. The deletion of *Atg7* under the control of the *Tyrosinase* promoter resulted in age-dependent changes of skin pigmentation (Zhang et al., 2015), accumulation of p62 in pigment cells of the eye (Sukseree et al., 2016), and aging-related p62 aggregation in neurons and neuroepithelial cells (Sukseree et al., 2018c). Atg7-deficient skin melanocytes developed premature senescence and showed a dysregulated antioxidant response and lipid homeostasis in culture (Zhang et al., 2015). We observed hyperinduction of the redox responsive Nrf2 system, probably due to accumulation of the autophagy adaptor and Nrf2 agonist p62, which did not prevent elevated melanocyte redox stress. The autophagy deficient melanocytes displayed a senescence associated secretory phenotype (SASP) (Ni et al., 2016). This low-grade inflammation is typical for senescent cells, and melanocyte SASP is implicated in the pathogenesis of vitiligo (Bellei et al., 2013; Qiao et al., 2016) and hypopigmented macules in tuberous sclerosis complex (Yang et al., 2018).

### Roles of Autophagy in Melanocyte Senescence, Proliferation, and Death

Several recent studies have established links between autophagy and the control of proliferation, senescence and death of melanocytes as well as melanoma cells. The autophagy gene *ATG5* is expressed at reduced levels in melanoma but not in benign melanocytes in nevi (Liu et al., 2013). In melanoma cells cultured *in vitro*, activation of *ATG5* reduced proliferation and induced senescence. The authors concluded that in early stages of melanoma development, inhibition of autophagy might delay the onset of oncogene-induced senescence and promote uncontrolled proliferation of melanocytes (Liu et al., 2013). Sample and colleagues reported that the autophagy adapter p62, which we had found to accumulate in melanocytes upon UVA exposure (Zhang et al., 2015), was elevated in abundance in both nevi and malignant melanoma, and proposed p62 as an oncogene in melanoma development (Sample et al., 2018). In a different study, pharmacological blockade of autophagy sensitized melanoma cells to chemotherapy induced cell death (Qiao et al., 2013). In contrast, starvation elevated both autophagy and cisplatin chemotoxicity in a murine model (Antunes et al., 2018). One of the possible connectors of autophagy, cell cycle control and aging in melanocytes and melanoma is the p53 activating protein ARF, which acts as emergency control of superoxide levels in mitochondrial dysfunction. This protein is rendered non-functional in suppressing superoxide and melanoma cell proliferation when *CDKN2A* is mutated (Christensen et al., 2014). ARF is however also an autophagy agonist (Balaburski et al., 2010), and this activity does not depend on the *CDKN2A* mutation. Whether modulation of autophagy would be an advisable strategy to promote the success of chemotherapy, or biologics will need further in-depth investigation (Li et al., 2019).

### Autophagy in Dermal Fibroblasts

The fibroblasts of the human dermis are long-lived cells and thus prone to accumulate intrinsic and extrinsic damage. Dermal fibroblasts do not represent a uniform population because they can take on at least two phenotypes, i.e., papillary and reticular (Driskell et al., 2013). The phenotypic plasticity of fibroblasts likely depends on the microenvironment (Korosec et al., 2019), in which the ECM is undergoes aging-related changes (Larroque-Cardoso et al., 2015). During skin aging the balance between these two fibroblast types shifts toward the reticular lineage phenotype (Mine et al., 2008).

Changes in autophagic flux in fibroblasts have been implicated in aging (Dumit et al., 2014; Demirovic et al., 2015; Kim et al., 2018). Recent findings discussed below suggest that autophagy and its age-related dysfunction may affect the microenvironment in chronologic and UV-induced aging as well as in premature aging phenotypes.

### Roles of Fibroblast Autophagy in Premature Aging and Age-Related Diseases

Cockayne syndrome is a premature aging disease affecting the skin and causing UV hypersensitivity and subcutaneous fat wasting. Fibroblasts from patients deficient in Cockayne syndrome B (CSB) protein display impaired autophagy due to interaction of CSB with HDAC6 and cytoskeletal components. Importantly, the restoration of autophagic function could revert the premature aging and photosensitivity phenotype in a CSB mouse model (Majora et al., 2018). The movement of the autophagic-lysosomal components along cytoskeletal components through motor proteins was demonstrated in a study in mouse fibroblasts. A genetic reduction of the motor protein KIF3C led to diminished autophagic flux and impaired proteostasis, similar in effect to a reduction of KIFC3 levels in naturally aged cells (Bejarano et al., 2018). Autophagy degraded nuclear anomalies resulting from mutation of lamin A (Park et al., 2009), and stimulation of autophagy and the connected Nrf2-mediated antioxidant response promote the clearance of the truncated pre-lamin A protein (progerin) which accumulates in fibroblasts of patients with Hutchinson Gilford progeria (Gabriel et al., 2017). Dermal fibroblasts from Parkinson’s disease patients displayed dysfunctional autophagy, mitochondrial damage, and impaired redox stress tolerance (Teves et al., 2018). The skin is a potential source of prognostic biomarkers for age-related neurodegenerative diseases (Can Akerman et al., 2019), and markers related to skin fibroblasts may also be relevant for disease-free skin aging.

### Roles of Autophagy in Proteostasis of Aging Fibroblasts

Loss of homeostasis in protein quality control – termed proteostasis – and thereby the accumulation of oxidized proteins is observed in the chronologic and photo-aging processes of the skin (Tigges et al., 2014). Proteostasis was identified as the ontology category predominantly affected by aging of human fibroblasts (Waldera-Lupa et al., 2014). Loss of proteostasis is attributed to dysregulation of the proteasome and autophagy in aging (Korovila et al., 2017). A comparison of proteostasis in phylogenetically related short- and long-lived mammalian species indicated higher autophagic activity in the longer lived species (Pride et al., 2015). In humans, loss of proteostasis has been observed in skin fibroblasts upon chronologic aging and UV exposure (Bulteau et al., 2007). The loss of capacity to remove oxidized and misfolded protein can lead to activation of DNA damage repair (DDR) pathways and thereby to the induction of cellular senescence (Chondrogianni and Gonos, 2008) and SASP (Catalgol and Grune, 2009).

Fibroblast autophagy is required for clearance of lipofuscin, an accumulation of misfolded and modified proteins and lipids (Höhn et al., 2012). Impairment of autophagy in senescent human fibroblasts (Ott et al., 2016) may thus be related to the aberrant deposition of lipofuscin that underlies age-related pigmentation irregularities. Declining autophagy in aging dermal fibroblasts was also implicated in another hallmark of skin aging, i.e., modifications of the ECM (Tashiro et al., 2014).

### Roles of Autophagy in the Control of DNA Damage in Fibroblasts

The function of autophagy in the protection against stress and damage was investigated in several studies on effects of ultraviolet irradiation of fibroblasts. UVB exposure, which promotes photoaging and senescence of dermal fibroblasts, induced proteasome inhibition and autophagy (Cavinato and Jansen-Dürr, 2017), whereas chronic UVA exposure led to blockage of autophagic flux (Lamore and Wondrak, 2013). *In vitro*, activation of autophagy by rapamycin prevented UVB induced senescence by limiting ROS production (Qin et al., 2018). Autophagy was proposed to be required for UV mediated senescence induction (Cavinato and Jansen-Dürr, 2017; Sample and He, 2017) but this hypothesis remains to be tested in different cells under different regimens of UV exposure. Current evidence suggests that autophagy counteracts the accumulation of damage that promotes cellular senescence.

## Conclusion

Skin aging is a complex process which, according to the concept described above, depends on changes in three categories of cells with differential roles of autophagy. Epithelial stem cells require autophagy for homeostasis during their long lifetime whereas short-lived differentiating epithelial cells utilize autophagy mainly for intracellular remodeling. Long-lived differentiated cells of both epithelial and non-epithelial origin need autophagy to suppress the accumulation of noxious compounds. We propose that inefficiencies of cellular processes (intrinsic drivers of aging) and damaging effects from the environment (extrinsic drivers of aging) compromise the machinery of autophagy, leading to a self-accelerating decline in cellular waste disposal and recycling in long-lived skin cells and decreasing protective functions of skin epithelia. Together, autophagy-dependent and independent processes lead to tissues changes that manifest in skin aging. Further characterization of autophagy in distinct skin cells may help to identify new approaches for maintaining the normal function of the skin in aged individuals.

## Author Contributions

LE, ET, and FG wrote the manuscript.

## Conflict of Interest Statement

The authors declare that the research was conducted in the absence of any commercial or financial relationships that could be construed as a potential conflict of interest.

## References

[B1] Akinduro O. Sully K. Patel A. Robinson D. J. Chikh A. McPhail G. (2016). Constitutive autophagy and nucleophagy during epidermal differentiation. *J. Invest. Dermatol.* 136 1460–1470. 10.1016/j.jid.2016.03.016 27021405

[B2] Antunes F. Pereira G. J. Jasiulionis M. G. Bincoletto C. Smaili S. S. (2018). Nutritional shortage augments cisplatin-effects on murine melanoma cells. *Chem. Biol. Interact.* 281 89–97. 10.1016/j.cbi.2017.12.027 29273566

[B3] Balaburski G. M. Hontz R. D. Murphy M. E. (2010). p53 and ARF: unexpected players in autophagy. *Trends Cell Biol.* 20 363–369. 10.1016/j.tcb.2010.02.007 20303758 PMC2891045

[B4] Bejarano E. Murray J. W. Wang X. Pampliega O. Yin D. Patel B. (2018). Defective recruitment of motor proteins to autophagic compartments contributes to autophagic failure in aging. *Aging Cell* 17:e12777. 10.1111/acel.12777 29845728 PMC6052466

[B5] Bellei B. Pitisci A. Ottaviani M. Ludovici M. Cota C. Luzi F. (2013). Vitiligo: a possible model of degenerative diseases. *PLoS One* 8:e59782. 10.1371/journal.pone.0059782 23555779 PMC3608562

[B6] Botchkarev V. A. (2017). Second international symposium-Epigenetic regulation of skin regeneration and aging: from chromatin biology towards the understanding of epigenetic basis of skin diseases. *J. Invest. Dermatol.* 137 1604–1608. 10.1016/j.jid.2017.01.037 28583676

[B7] Boya P. Codogno P. Rodriguez-Muela N. (2018). Autophagy in stem cells: repair, remodelling and metabolic reprogramming. *Development* 145:dev146506. 10.1242/dev.146506 29483129

[B8] Boya P. Reggiori F. Codogno P. (2013). Emerging regulation and functions of autophagy. *Nat. Cell Biol.* 15 713–720. 10.1038/ncb2788 23817233 PMC7097732

[B9] Bruckner-Tuderman L. (2012). “Biology of the extracellular matrix,” in *Dermatology*, eds Bolognia J. L. Jorizzl J. L. Schaffer J. V. (St. Louis, MO: Elsevier), 1585–1598.

[B10] Bulteau A. L. Moreau M. Nizard C. Friguet B. (2007). Proteasome and photoaging: the effects of UV irradiation. *Ann. N. Y. Acad. Sci.* 1100 280–290. 10.1196/annals.1395.029 17460189

[B11] Can Akerman S. Hossain S. Shobo A. Zhong Y. Jourdain R. Hancock M. (2019). Neurodegenerative disease-related proteins within the epidermal layer of the human skin. *J. Alzheimers Dis.* 69 463–478. 10.3233/JAD-181191 31006686

[B12] Catalgol B. Grune T. (2009). Protein pool maintenance during oxidative stress. *Curr. Pharm. Des.* 15 3043–3051. 10.2174/138161209789058129 19754378

[B13] Cavinato M. Jansen-Dürr P. (2017). Molecular mechanisms of UVB-induced senescence of dermal fibroblasts and its relevance for photoaging of the human skin. *Exp. Gerontol.* 94 78–82. 10.1016/j.exger.2017.01.009 28093316

[B14] Cawley E. P. Hsu Y. T. Sturgill B. C. Harman L. E. Jr. (1973). Lipofuscin (“wear and tear pigment”) in human sweat glands. *J. Invest. Dermatol.* 61 105–107. 10.1111/1523-1747.ep12675428 4125553

[B15] Chai M. Jiang M. Vergnes L. Fu X. de Barros S. C. Doan N. B. (2019). Stimulation of hair growth by small molecules that activate autophagy. *Cell Rep.* 27 3413–3421. 10.1016/j.celrep.2019.05.070 31216464

[B16] Chang A. L. S. (2016). Expanding our understanding of human skin aging. *J. Invest. Dermatol.* 136 897–899. 10.1016/j.jid.2016.02.020 27107374

[B17] Cho I. J. Lui P. P. Obajdin J. Riccio F. Stroukov W. Willis T. L. (2019). Mechanisms, hallmarks, and implications of stem cell quiescence. *Stem Cell Rep.* 12 1190–1200. 10.1016/j.stemcr.2019.05.012 31189093 PMC6565921

[B18] Chondrogianni N. Gonos E. S. (2008). Proteasome activation as a novel antiaging strategy. *IUBMB Life* 60 651–655. 10.1002/iub.99 18506854

[B19] Christensen C. Bartkova J. Mistrík M. Hall A. Lange M. K. Ralfkiær U. (2014). A short acidic motif in ARF guards against mitochondrial dysfunction and melanoma susceptibility. *Nat. Commun.* 5:5348. 10.1038/ncomms6348 25370744

[B20] Cullup T. Kho A. L. Dionisi-Vici C. Brandmeier B. Smith F. Urry Z. (2013). Recessive mutations in EPG5 cause Vici syndrome, a multisystem disorder with defective autophagy. *Nat. Genet.* 45 83–87. 10.1038/ng.2497 23222957 PMC4012842

[B21] Damsky W. E. Bosenberg M. (2017). Melanocytic nevi and melanoma: unraveling a complex relationship. *Oncogene* 36 5771–5792. 10.1038/onc.2017.189 28604751 PMC5930388

[B22] Das L. M. Binko A. M. Traylor Z. P. Peng H. Lu K. Q. (2019). Vitamin D improves sunburns by increasing autophagy in M2 macrophages. *Autophagy* 15 813–826. 10.1080/15548627.2019.1569298 30661440 PMC6526871

[B23] Demirovic D. Nizard C. Rattan S. I. (2015). Basal level of autophagy is increased in aging human skin fibroblasts in vitro, but not in old skin. *PLoS One* 10:e0126546. 10.1371/journal.pone.0126546 25950597 PMC4423894

[B24] Deruy E. Nassour J. Martin N. Vercamer C. Malaquin N. Bertout J. (2014). Level of macroautophagy drives senescent keratinocytes into cell death or neoplastic evasion. *Cell Death Dis.* 5:e1577. 10.1038/cddis.2014.533 25522271 PMC4649843

[B25] Driskell R. R. Lichtenberger B. M. Hoste E. Kretzschmar K. Simons B. D. Charalambous M. (2013). Distinct fibroblast lineages determine dermal architecture in skin development and repair. *Nature* 504 277–281. 10.1038/nature12783 24336287 PMC3868929

[B26] Dufour A. Candas V. (2007). Aging and thermal responses during passive heat exposure: sweating and sensory aspects. *Eur. J. Appl. Physiol.* 100 19–26. 10.1007/s00421-007-0396-9 17242944

[B27] Dumit V. I. Küttner V. Käppler J. Piera-Velazquez S. Jimenez S. A. Bruckner-Tuderman L. (2014). Altered MCM protein levels and autophagic flux in aged and systemic sclerosis dermal fibroblasts. *J. Invest. Dermatol.* 134 2321–2330. 10.1038/jid.2014.69 24496236 PMC4121389

[B28] Eckhart L. Lippens S. Tschachler E. Declercq W. (2013). Cell death by cornification. *Biochim. Biophys. Acta* 1833 3471–3480. 10.1016/j.bbamcr.2013.06.010 23792051

[B29] Eckhart L. Zeeuwen P. L. J. M. (2018). The skin barrier: epidermis versus environment. *Exp. Dermatol.* 27 805–806. 10.1111/exd.13731 29989217

[B30] Feng J. Luo J. Yang P. Du J. Kim B. S. Hu H. (2018). Piezo2 channel-Merkel cell signaling modulates the conversion of touch to itch. *Science* 360 530–533. 10.1126/science.aar5703 29724954 PMC6114129

[B31] Fernandez-Flores A. Saeb-Lima M. Cassarino D. S. (2019). Histopathology of aging of the hair follicle. *J. Cutan. Pathol.* 10.1111/cup.13467. [Epub ahead of print]. 30932205

[B32] Fischer H. Fumicz J. Rossiter H. Napirei M. Buchberger M. Tschachler E. (2017). Holocrine secretion of sebum is a unique DNase2-dependent mode of programmed cell death. *J. Invest. Dermatol.* 137 587–594. 10.1016/j.jid.2016.10.017 27771328

[B33] Fisher G. J. Wang Z. Q. Datta S. C. Varani J. Kang S. Voorhees J. J. (1997). Pathophysiology of premature skin aging induced by ultraviolet light. *N. Engl. J. Med.* 337 1419–1428. 9358139 10.1056/NEJM199711133372003

[B34] Gabriel D. Shafry D. D. Gordon L. B. Djabali K. (2017). Intermittent treatment with farnesyltransferase inhibitor and sulforaphane improves cellular homeostasis in Hutchinson-Gilford progeria fibroblasts. *Oncotarget* 8 64809–64826. 10.18632/oncotarget.19363 29029393 PMC5630293

[B35] García-Prat L. Martínez-Vicente M. Perdiguero E. Ortet L. Rodríguez-Ubreva J. Rebollo E. (2016). Autophagy maintains stemness by preventing senescence. *Nature* 529 37–42. 10.1038/nature16187 26738589

[B36] Galluzzi L. Baehrecke E. H. Ballabio A. Boya P. Bravo-San Pedro J. M. Cecconi F. (2017). Molecular definitions of autophagy and related processes. *EMBO J.* 36 1811–1836. 10.15252/embj.201796697 28596378 PMC5494474

[B37] Ghosh K. Capell B. C. (2016). The senescence-associated secretory phenotype: critical effector in skin cancer and aging. *J. Invest. Dermatol.* 136 2133–2139. 10.1016/j.jid.2016.06.621 27543988 PMC5526201

[B38] Gilchrest B. A. (2013). Photoaging. *J. Invest. Dermatol.* 133 E2–E6. 10.1038/skinbio.2013.176 23820721

[B39] Gilchrest B. A. Krutmann J. (2006). *Skin Aging.* Berlin: Springer-Verlag.

[B40] Guinot C. Malvy D. J. Ambroisine L. Latreille J. Mauger E. Tenenhaus M. (2002). Relative contribution of intrinsic vs extrinsic factors to skin aging as determined by a validated skin age score. *Arch. Dermatol.* 138 1454–1460. 12437451 10.1001/archderm.138.11.1454

[B41] Hansen M. Rubinsztein D. C. Walker D. W. (2018). Autophagy as a promoter of longevity: insights from model organisms. *Nat. Rev. Mol. Cell Biol.* 19 579–593. 10.1038/s41580-018-0033-y 30006559 PMC6424591

[B42] Haruna K. Suga Y. Muramatsu S. Taneda K. Mizuno Y. Ikeda S. (2008). Differentiation-specific expression and localization of an autophagosomal marker protein (LC_3_) in human epidermal keratinocytes. *J. Dermatol. Sci.* 52 213–215. 10.1016/j.jdermsci.2008.07.005 18760570

[B43] Höhn A. Sittig A. Jung T. Grimm S. Grune T. (2012). Lipofuscin is formed independently of macroautophagy and lysosomal activity in stress-induced prematurely senescent human fibroblasts. *Free Radic. Biol. Med.* 53 1760–1769. 10.1016/j.freeradbiomed.2012.08.591 22982048

[B44] Itou T. (2018). Morphological changes in hair melanosomes by aging. *Pigment Cell Melanoma Res.* 31 630–635. 10.1111/pcmr.12697 29488689

[B45] Jaeger K. Sukseree S. Zhong S. Phinney B. S. Mlitz V. Buchberger M. (2019). Cornification of nail keratinocytes requires autophagy for bulk degradation of intracellular proteins while sparing components of the cytoskeleton. *Apoptosis* 24 62–73. 10.1007/s10495-018-1505-4 30552537 PMC6373260

[B46] Jonason A. S. Kunala S. Price G. J. Restifo R. J. Spinelli H. M. Persing J. A. (1996). Frequent clones of p53-mutated keratinocytes in normal human skin. *Proc. Natl. Acad. Sci. U.S.A.* 93 14025–14029. 10.1073/pnas.93.24.14025 8943054 PMC19488

[B47] Kim H. S. Park S. Y. Moon S. H. Lee J. D. Kim S. (2018). Autophagy in human skin fibroblasts: impact of age. *Int. J. Mol. Sci.* 19:E2254. 10.3390/ijms19082254 30071626 PMC6121946

[B48] Kligman A. M. Balin A. K. (1989). “Aging of human skin,” in *Aging and the Skin*, eds Balin A. K. Kligman A. M. (New York, NY: Raven Press).

[B49] Korosec A. Frech S. Gesslbauer B. Vierhapper M. Radtke C. Petzelbauer P. (2019). Lineage identity and location within the dermis determine the function of papillary and reticular fibroblasts in human skin. *J. Invest. Dermatol.* 139 342–351. 10.1016/j.jid.2018.07.033 30179601

[B50] Korovila I. Hugo M. Castro J. P. Weber D. Höhn A. Grune T. (2017). Proteostasis, oxidative stress and aging. *Redox Biol.* 13 550–567. 10.1016/j.redox.2017.07.008 28763764 PMC5536880

[B51] Lamore S. D. Wondrak G. T. (2013). UVA causes dual inactivation of cathepsin B and L underlying lysosomal dysfunction in human dermal fibroblasts. *J. Photochem. Photobiol. B* 123 1–12. 10.1016/j.jphotobiol.2013.03.007 23603447 PMC3710731

[B52] Larroque-Cardoso P. Camaré C. Nadal-Wollbold F. Grazide M. H. Pucelle M. Garoby-Salom S. (2015). Elastin modification by 4-hydroxynonenal in hairless mice exposed to UV-A. Role in photoaging and actinic elastosis. *J. Invest. Dermatol.* 135 1873–1881. 10.1038/jid.2015.84 25739050

[B53] Leidal A. M. Levine B. Debnath J. (2018). Autophagy and the cell biology of age-related disease. *Nat. Cell Biol.* 20 1338–1348. 10.1038/s41556-018-0235-8 30482941

[B54] Levine B. Kroemer G. (2019). Biological functions of autophagy genes: a disease perspective. *Cell* 176 11–42. 10.1016/j.cell.2018.09.048 30633901 PMC6347410

[B55] Li S. Song Y. Quach C. Guo H. Jang G. B. Maazi H. (2019). Transcriptional regulation of autophagy-lysosomal function in BRAF-driven melanoma progression and chemoresistance. *Nat. Commun.* 10:1693. 10.1038/s41467-019-09634-8 30979895 PMC6461621

[B56] Liu H. He Z. von Rütte T. Yousefi S. Hunger R. E. Simon H. U. (2013). Down-regulation of autophagy-related protein 5 (ATG5) contributes to the pathogenesis of early-stage cutaneous melanoma. *Sci. Transl. Med.* 5:202ra123. 10.1126/scitranslmed.3005864 24027027

[B57] Liu N. Matsumura H. Kato T. Ichinose S. Takada A. Namiki T. (2019). Stem cell competition orchestrates skin homeostasis and ageing. *Nature* 568 344–350. 10.1038/s41586-019-1085-7 30944469

[B58] López-Otín C. Blasco M. A. Partridge L. Serrano M. Kroemer G. (2013). The hallmarks of aging. *Cell* 153 1194–1217. 10.1016/j.cell.2013.05.039 23746838 PMC3836174

[B59] Lu C. Fuchs E. (2014). Sweat gland progenitors in development, homeostasis, and wound repair. *Cold Spring Harb. Perspect. Med.* 4:a015222. 10.1101/cshperspect.a015222 24492848 PMC3904096

[B60] Mahil S. K. Twelves S. Farkas K. Setta-Kaffetzi N. Burden A. D. Gach J. E. (2016). AP1S3 mutations cause skin autoinflammation by disrupting keratinocyte autophagy and up-regulating IL-36 production. *J. Invest. Dermatol.* 136 2251–2259. 10.1016/j.jid.2016.06.618 27388993 PMC5070969

[B61] Majora M. Sondenheimer K. Knechten M. Uthe I. Esser C. Schiavi A. (2018). HDAC inhibition improves autophagic and lysosomal function to prevent loss of subcutaneous fat in a mouse model of Cockayne syndrome. *Sci. Transl. Med.* 10:eaam7510. 10.1126/scitranslmed.aam7510 30158153

[B62] Matsumura H. Mohri Y. Binh N. T. Morinaga H. Fukuda M. Ito M. (2016). Hair follicle aging is driven by transepidermal elimination of stem cells via COL17A1 proteolysis. *Science* 351:aad4395. 10.1126/science.aad4395 26912707

[B63] McGlone F. Reilly D. (2010). The cutaneous sensory system. *Neurosci. Biobehav. Rev.* 34 148–159. 10.1016/j.neubiorev.2009.08.004 19712693

[B64] McGrath J. A. (2005). “The structure and function of skin,” in *Pathology of the Skin*, eds McKee P. H. Calonje E. Granter S. R. Brann T. (St Louis, MO: Elsevier), 1–36.

[B65] Mine S. Fortunel N. O. Pageon H. Asselineau D. (2008). Aging alters functionally human dermal papillary fibroblasts but not reticular fibroblasts: a new view of skin morphogenesis and aging. *PLoS One* 3:e4066. 10.1371/journal.pone.0004066 19115004 PMC2605251

[B66] Muñoz-Espín D. Serrano M. (2014). Cellular senescence: from physiology to pathology. *Nat. Rev. Mol. Cell Biol.* 15 482–496. 10.1038/nrm3823 24954210

[B67] Murase D. Hachiya A. Fullenkamp R. Beck A. Moriwaki S. Hase T. (2016). Variation in Hsp70-1A expression contributes to skin color diversity. *J. Invest. Dermatol.* 136 1681–1691. 10.1016/j.jid.2016.03.038 27094592 PMC5584801

[B68] Murase D. Hachiya A. Takano K. Hicks R. Visscher M. O. Kitahara T. (2013). Autophagy has a significant role in determining skin color by regulating melanosome degradation in keratinocytes. *J. Invest. Dermatol.* 133 2416–2424. 10.1038/jid.2013.165 23558403

[B69] Ni C. Narzt M. S. Nagelreiter I. M. Zhang C. F. Larue L. Rossiter H. (2016). Autophagy deficient melanocytes display a senescence associated secretory phenotype that includes oxidized lipid mediators. *Int. J. Biochem. Cell Biol.* 81(Pt B), 375–382. 10.1016/j.biocel.2016.10.006 27732890

[B70] Nicu C. Hardman J. A. Pople J. Paus R. (2019). Do human dermal adipocytes switch from lipogenesis in anagen to lipophagy and lipolysis during catagen in the human hair cycle? *Exp. Dermatol.* 28 432–435. 10.1111/exd.13904 30776154

[B71] Ott C. König J. Höhn A. Jung T. Grune T. (2016). Macroautophagy is impaired in old murine brain tissue as well as in senescent human fibroblasts. *Redox Biol.* 10 266–273. 10.1016/j.redox.2016.10.015 27825071 PMC5099282

[B72] Ovadya Y. Landsberger T. Leins H. Vadai E. Gal H. Biran A. (2018). Impaired immune surveillance accelerates accumulation of senescent cells and aging. *Nat. Commun.* 9:5435. 10.1038/s41467-018-07825-3 30575733 PMC6303397

[B73] Park Y. E. Hayashi Y. K. Bonne G. Arimuram T. Noguchim S. Nonakam I. (2009). Autophagic degradation of nuclear components in mammalian cells. *Autophagy* 5 795–804. 10.4161/auto.8901 19550147

[B74] Parodi C. Hardman J. A. Allavena G. Marotta R. Catelani T. Bertolini M. (2018). Autophagy is essential for maintaining the growth of a human (mini-)organ: evidence from scalp hair follicle organ culture. *PLoS Biol.* 16:e2002864. 10.1371/journal.pbio.2002864 29590104 PMC5891029

[B75] Pride H. Yu Z. Sunchu B. Mochnick J. Coles A. Zhang Y. (2015). Long-lived species have improved proteostasis compared to phylogenetically-related shorter-lived species. *Biochem. Biophys. Res. Commun.* 457 669–675. 10.1016/j.bbrc.2015.01.046 25615820

[B76] Qiang L. Sample A. Shea C. R. Soltani K. Macleod K. F. He Y. Y. (2017). Autophagy gene ATG7 regulates ultraviolet radiation-induced inflammation and skin tumorigenesis. *Autophagy* 13 2086–2103. 10.1080/15548627.2017.1380757 28933598 PMC5788558

[B77] Qiang L. Wu C. Ming M. Viollet B. He Y. Y. (2013). Autophagy controls p38 activation to promote cell survival under genotoxic stress. *J. Biol. Chem.* 288 1603–1611. 10.1074/jbc.M112.415224 23212914 PMC3548470

[B78] Qiang L. Zhao B. Shah P. Sample A. Yang S. He Y. Y. (2016). Autophagy positively regulates DNA damage recognition by nucleotide excision repair. *Autophagy* 12 357–368. 10.1080/15548627.2015.1110667 26565512 PMC4835978

[B79] Qiao S. Tao S. Rojo de la Vega M. Park S. L. Vonderfecht A. A. Jacobs S. L. (2013). The antimalarial amodiaquine causes autophagic-lysosomal and proliferative blockade sensitizing human melanoma cells to starvation- and chemotherapy-induced cell death. *Autophagy* 9 2087–2102. 10.4161/auto.26506 24113242 PMC3981748

[B80] Qiao Z. Wang X. Xiang L. Zhang C. (2016). Dysfunction of autophagy: a possible mechanism involved in the pathogenesis of vitiligo by breaking the redox balance of melanocytes. *Oxid. Med. Cell. Longev.* 2016:3401570. 10.1155/2016/3401570 28018522 PMC5153471

[B81] Qin D. Ren R. Jia C. Lu Y. Yang Q. Chen L. (2018). Rapamycin protects skin fibroblasts from ultraviolet B-induced photoaging by suppressing the production of reactive oxygen species. *Cell Physiol. Biochem.* 46 1849–1860. 10.1159/000489369 29705807

[B82] Ramkumar A. Murthy D. Raja D. A. Singh A. Krishnan A. Khanna S. (2017). Classical autophagy proteins LC3B and ATG4B facilitate melanosome movement on cytoskeletal tracks. *Autophagy* 13 1331–1347. 10.1080/15548627.2017.1327509 28598240 PMC5584859

[B83] Rinnerthaler M. Richter K. (2018). The influence of calcium on the skin pH and epidermal barrier during aging. *Curr. Probl. Dermatol.* 54 79–86. 10.1159/000489521 30130776

[B84] Rossiter H. König U. Barresi C. Buchberger M. Ghannadan M. Zhang C. F. (2013). Epidermal keratinocytes form a functional skin barrier in the absence of Atg7 dependent autophagy. *J. Dermatol. Sci.* 71 67–75. 10.1016/j.jdermsci.2013.04.015 23669018

[B85] Rossiter H. Stübiger G. Gröger M. König U. Gruber F. Sukseree S. (2018). Inactivation of autophagy leads to changes in sebaceous gland morphology and function. *Exp. Dermatol.* 27 1142–1151. 10.1111/exd.13752 30033522

[B86] Rubinsztein D. C. Mariño G. Kroemer G. (2011). Autophagy and aging. *Cell* 146 682–695. 21884931 10.1016/j.cell.2011.07.030

[B87] Sample A. He Y. Y. (2017). Autophagy in UV damage response. *Photochem. Photobiol.* 93 943–955. 10.1111/php.12691 27935061 PMC5466513

[B88] Sample A. Zhao B. Qiang L. He Y. Y. (2017). Adaptor protein p62 promotes skin tumor growth and metastasis and is induced by UVA radiation. *J. Biol. Chem.* 292 14786–14795. 10.1074/jbc.M117.786160 28724634 PMC5592660

[B89] Sample A. Zhao B. Wu C. Qian S. Shi X. Aplin A. (2018). The autophagy receptor adaptor p62 is up-regulated by UVA radiation in melanocytes and in melanoma cells. *Photochem. Photobiol.* 94 432–437. 10.1111/php.12809 28715145 PMC5771989

[B90] Schäfer T. Merkl J. Klemm E. Wichmann H. E. Ring J. Kora Study Group (2006). The epidemiology of nevi and signs of skin aging in the adult general population: results of the KORA-survey 2000. *J. Invest. Dermatol.* 126 1490–1496. 10.1038/sj.jid.5700269 16645597

[B91] Settembre C. Cinque L. Bartolomeo R. Di Malta C. De Leonibus C. Forrester A. (2018). Defective collagen proteostasis and matrix formation in the pathogenesis of lysosomal storage disorders. *Matrix Biol.* 71–72 283–293. 10.1016/j.matbio.2018.06.001 29870768

[B92] Sil P. Wong S. W. Martinez J. (2018). More than skin deep: autophagy is vital for skin barrier function. *Front. Immunol.* 9:1376. 10.3389/fimmu.2018.01376 29988591 PMC6026682

[B93] Solanas G. Peixoto F. O. Perdiguero E. Jardí M. Ruiz-Bonilla V. Datta D. (2017). Aged stem cells reprogram their daily rhythmic functions to adapt to stress. *Cell* 170 678–692. 10.1016/j.cell.2017.07.035 28802040

[B94] Song X. Narzt M. S. Nagelreiter I. M. Hohensinner P. Terlecki-Zaniewicz L. Tschachler E. (2017). Autophagy deficient keratinocytes display increased DNA damage, senescence and aberrant lipid composition after oxidative stress in vitro and in vivo. *Redox Biol.* 11 219–230. 10.1016/j.redox.2016.12.015 28012437 PMC5192251

[B95] Sukseree S. Bergmann S. Pajdzik K. Sipos W. Gruber F. Tschachler E. (2018a). Suppression of epithelial autophagy compromises the homeostasis of sweat glands during aging. *J. Invest. Dermatol.* 138 2061–2063. 10.1016/j.jid.2018.03.1502 29571941

[B96] Sukseree S. Bergmann S. Pajdzik K. Tschachler E. Eckhart L. (2018b). Suppression of autophagy perturbs turnover of sequestosome-1/p62 in Merkel cells but not in keratinocytes. *J. Dermatol. Sci.* 90 209–211. 10.1016/j.jdermsci.2018.01.008 29395578

[B97] Sukseree S. László L. Gruber F. Bergmann S. Narzt M. S. Nagelreiter I. M. (2018c). Filamentous aggregation of sequestosome-1/p62 in brain neurons and neuroepithelial cells upon Tyr-Cre-mediated deletion of the autophagy gene Atg7. *Mol. Neurobiol.* 55 8425–8437. 10.1007/s12035-018-0996-x 29550918 PMC6153718

[B98] Sukseree S. Chen Y. T. Laggner M. Gruber F. Petit V. Nagelreiter I. M. (2016). Tyrosinase-Cre-mediated deletion of the autophagy gene Atg7 leads to accumulation of the RPE65 variant M450 in the retinal pigment epithelium of C57BL/6 mice. *PLoS One* 11:e0161640. 10.1371/journal.pone.0161640 27537685 PMC4990303

[B99] Sukseree S. Eckhart L. Tschachler E. Watanapokasin R. (2013a). Autophagy in epithelial homeostasis and defense. *Front. Biosci.* 5 1000–1010. 10.2741/e679 23747915

[B100] Sukseree S. Rossiter H. Mildner M. Pammer J. Buchberger M. Gruber F. (2013b). Targeted deletion of Atg5 reveals differential roles of autophagy in keratin K5-expressing epithelia. *Biochem. Biophys. Res. Commun.* 430 689–694. 10.1016/j.bbrc.2012.11.090 23211599

[B101] Sukseree S. Mildner M. Rossiter H. Pammer J. Zhang C.-F. Watanapokasin R. (2012). Autophagy in the thymic epithelium is dispensable for the development of self-tolerance in a novel mouse model. *PLoS One* 7:e38933. 10.1371/journal.pone.0038933 22719991 PMC3377705

[B102] Tashiro K. Shishido M. Fujimoto K. Hirota Y. Yo K. Gomi T. (2014). Age-related disruption of autophagy in dermal fibroblasts modulates extracellular matrix components. *Biochem. Biophys. Res. Commun.* 443 167–172. 10.1016/j.bbrc.2013.11.066 24287182

[B103] Tekirdag K. Cuervo A. M. (2018). Chaperone-mediated autophagy and endosomal microautophagy: joint by a chaperone. *J. Biol. Chem.* 293 5414–5424. 10.1074/jbc.R117.818237 29247007 PMC5900761

[B104] Teves J. M. Y. Bhargava V. Kirwan K. R. Corenblum M. J. Justiniano R. Wondrak G. T. (2018). Parkinson’s disease skin fibroblasts display signature alterations in growth, redox homeostasis, mitochondrial function, and autophagy. *Front. Neurosci.* 11:737. 10.3389/fnins.2017.00737 29379409 PMC5770791

[B105] Tigges J. Krutmann J. Fritsche E. Haendeler J. Schaal H. Fischer J. W. (2014). The hallmarks of fibroblast ageing. *Mech. Ageing Dev.* 138 26–44. 10.1016/j.mad.2014.03.004 24686308

[B106] Tobin D. J. (2015). Age-related hair pigment loss. *Curr. Probl. Dermatol.* 47 128–138. 10.1159/000369413 26370651

[B107] Tran S. Rizos H. (2013). Human nevi lack distinguishing senescence traits. *Aging* 5 98–99. 10.18632/aging.100537 23518674 PMC3616196

[B108] Tschachler E. Eckhart L. (2017). Autophagy – how to control your intracellular diet. *Br. J. Dermatol.* 176 1417–1419. 10.1111/bjd.15566 28581245

[B109] Velarde M. C. (2017). Epidermal barrier protects against age-associated systemic inflammation. *J. Invest. Dermatol.* 137 1206–1208. 10.1016/j.jid.2017.02.964 28395899

[B110] Waldera-Lupa D. M. Kalfalah F. Florea A. M. Sass S. Kruse F. Rieder V. (2014). Proteome-wide analysis reveals an age-associated cellular phenotype of in situ aged human fibroblasts. *Aging* 6 56–78. 10.18632/aging.100698 25411231 PMC4247387

[B111] Wang A. S. Dreesen O. (2018). Biomarkers of cellular senescence and skin aging. *Front. Genet.* 9:247. 10.3389/fgene.2018.00247 30190724 PMC6115505

[B112] Wang Q. Bu S. Xin D. Li B. Wang L. Lai D. (2018). Autophagy is indispensable for the self-renewal and quiescence of ovarian cancer spheroid cells with stem cell-like properties. *Oxid. Med. Cell. Longev.* 2018:7010472. 10.1155/2018/7010472 30319732 PMC6167563

[B113] Yaar M. Gilchrest B. A. (1998). Aging versus photoaging: postulated mechanisms and effectors. *J. Investig. Dermatol. Symp. Proc.* 3 47–51. 10.1038/jidsymp.1998.12 9732058

[B114] Yaar M. Gilchrest B. A. (2001). Ageing and photoageing of keratinocytes and melanocytes. *Clin. Exp. Dermatol.* 26 583–591. 10.1046/j.1365-2230.2001.00895.x 11696062

[B115] Yang F. Yang L. Wataya-Kaneda M. Hasegawa J. Yoshimori T. Tanemura A. (2018). Dysregulation of autophagy in melanocytes contributes to hypopigmented macules in tuberous sclerosis complex. *J. Dermatol. Sci.* 89 155–164. 10.1016/j.jdermsci.2017.11.002 29146131

[B116] Yoshihara N. Ueno T. Takagi A. Oliva Trejo J. A. Haruna K. Suga Y. (2015). The significant role of autophagy in the granular layer in normal skin differentiation and hair growth. *Arch. Dermatol. Res.* 307 159–169. 10.1007/s00403-014-1508-0 25288166

[B117] Zalaudek I. Grinschgl S. Argenziano G. Marghoob A. A. Blum A. Richtig E. (2006). Age-related prevalence of dermoscopy patterns in acquired melanocytic naevi. *Br. J. Dermatol.* 154 299–304. 10.1111/j.1365-2133.2005.06973.x 16433800

[B118] Zhang C. F. Gruber F. Ni C. Mildner M. Koenig U. Karner S. (2015). Suppression of autophagy dysregulates the antioxidant response and causes premature senescence of melanocytes. *J. Invest. Dermatol.* 135 1348–1357. 10.1038/jid.2014.439 25290687

[B119] Zhao Y. Zhang C. F. Rossiter H. Eckhart L. König U. Karner S. (2013). Autophagy is induced by UVA and promotes removal of oxidized phospholipids and protein aggregates in epidermal keratinocytes. *J. Invest. Dermatol.* 133 1629–1637. 10.1038/jid.2013.26 23340736

